# The impact of personal medical savings accounts on healthcare utilization and out-of-pocket costs in public basic health insurance: a national cross-sectional study

**DOI:** 10.3389/fpubh.2025.1571746

**Published:** 2025-04-30

**Authors:** Jinming Yang, Wenjuan Tao, Xing Qu

**Affiliations:** ^1^West China Biomedical Big Data Center, West China Hospital, Sichuan University, Chengdu, China; ^2^Med-X Center for Informatics, Sichuan University, Chengdu, China; ^3^Institute of Hospital Management, West China Hospital, Sichuan University, Chengdu, China

**Keywords:** personal medical savings account, China basic medical insurance, health care utilization, out-of-pocket medical expenditure, insurance effect

## Abstract

**Objectives:**

Previous research has shown that the implementation of personal medical savings accounts in health insurance can impact health utilization and medical cost through enhancing individual responsibility for their own health. The aim of this study was to investigate whether there are differences in various healthcare utilization and out-of-pocket payment ratios between insured individuals with and without personal medical savings accounts in the context of basic medical insurance in China.

**Methods:**

A nationally representative cross-sectional data from the China Health and Retirement Longitudinal Study was used to analyze. Seemingly Unrelated Regression analysis was conducted to examine the potential impact of personal medical savings accounts on the utilization rates and self-payment ratios for outpatient services, hospitalization, dental treatment, and physical examinations. Heckman selection model was used as a sensitivity analysis to test the robust of original results.

**Results:**

A total of 15,628 individuals were included in the analysis. Among them, 95.5% were covered by basic medical insurance, while only 12.8% had a personal medical savings account. Possessing a personal medical savings account was significantly associated with increased utilization of dental services (OR: 1.327, 95% CI: 1.05–1.67) and a higher frequency of physical examinations (OR: 2.271, 95% CI: 1.86–2.77). This effect was not significant in outpatient and inpatient health service utilization. Furthermore, having a personal medical savings account was significantly associated with lower out-of-pocket payment ratios across various healthcare services. Specifically, individuals with such accounts experienced a 15.8 percentage point reduction in outpatient services, a 22.1 percentage point reduction in inpatient services, and a 13.4 percentage point reduction in dental services.

**Conclusion:**

The study revealed disparities between individuals with and without personal medical savings accounts in China. While these accounts can cover the insurer’s regular medical expenses, their effect on high-cost expenses appears to be limited. These findings suggest that reforms in medical insurance should focus on reducing the gap between insured individuals with and without personal medical savings accounts.

## Introduction

Basic health care insurance constitutes a vital element of the Universal Health Coverage advocated by the World Health Organization (WHO) (1). Research affirms that healthcare insurance can enhance healthcare accessibility, facilitate favorable health outcomes, encompassing an individual’s perception of their own health and well-being, incentivize judicious utilization of healthcare resources, and alleviate financial burden on individuals, families, and communities (2, 3).

In developing nations, achieving universal health insurance coverage remains a long-term objective (4). Remarkably, China has made significant strides in expanding both the scale and pace of coverage growth. By 2011, an impressive 95% of the Chinese population had obtained insurance, a stark increase from less than 50% in 2005 (5). This expansion primarily stems from two public insurance programs. URRBMI provides coverage for the unemployed, children, students, and the disabled across both rural and urban areas. This program resulted from the consolidation of the New Cooperative Medical Scheme (NCMS), launched in 1998 for the rural population, and the Urban Residents’ Basic Medical Insurance System (URBMI), introduced in 2007.

Secondly, the Urban Employee Basic Medical Insurance (UEBMI), instated in 1998 as an employment-based insurance initiative, encompasses coverage for the employed. Presently, China’s health insurance reaches a staggering 95% of the national population, encompassing over 1.34 billion individuals as of 2023. Within this demographic, URBMI caters to 72.2% of the insured populace, while the UEBMI and the other types of medical insurance accounts for the remaining 27.8% (6).

While significant progress has been made in enhancing insurance coverage in China, disparities persist between URBMI and UEBMI concerning funding resources, target demographics, reimbursement rates, and extent of coverage (7). These discrepancies contribute to variations in healthcare accessibility and financial burden for insured individuals (8, 9). Previous research has revealed that the funding scale for URBMI may fall short in meeting the escalating healthcare needs of the insured, as indicated by public financing data (10). In contrast, UEBMI, tailored for urban employed individuals, operates with higher funding thresholds and reimbursement rates (11, 12). As a result, a stratified system emerges, wherein individuals enrolled in urban health insurance are able to avail a broad range of high-quality services, while those enrolled in rural health insurance have access to a less comprehensive benefits package and face higher expenses due to co-payments or services that are not covered (13).

The difference mentioned above is rarely directly experienced at the individual level. However, there is a significant disparity between URBMI and UEBMI that can be easily observed and experienced by individual insurers. Unlike UEBMI, URBMI does not involve personal medical savings accounts. This disparity can even be subjectively felt within a household, particularly among unemployed and employed family members (14). The original intention of this model is to emphasize the mutual assistance of basic medical insurance, but also to strengthen the accumulation of medical insurance funds by enhancing personal responsibility and cost consciousness, and restrain the malignant inflation of medical expenses.

Similar to Medisave in Singapore (15), a personal medical savings account is a dedicated fund account established by the healthcare insurance institution for individuals participating in basic medical insurance. The individual account comprises 2% of individual wages and a portion contributed by the employer. It allows individuals to allocate a part of the amount for outpatient visits, medication purchases, part of dental care or other non-hospital medical expenses. Nine provinces have begun implementing the sharing of personal medical savings accounts within families, personal accounts can be used across provinces for their close relatives to pay resident medical insurance and reimburse medical expenses (16). Consequently, it may lead to variations in healthcare service utilization and expenditure between participants with or without medical savings accounts. However, rare prior studies have explored the impact of personal medical savings accounts on healthcare utilization in basic medical insurance in China.

On the perspective of medical insurance effect on health care utilization, the majority of prior studies predominantly focused on utilization of outpatient, inpatient or emergency services (17, 18), which was insufficient to elucidate the various healthcare modalities comprehensively. Regrettably, dental care, despite being a prevalent health issue in China as in many other nations, has often been overlooked in these studies (19). Dental diseases are among the most widespread health conditions in China (20); however, rare information exists regarding the correlation between dental care utilization and basic medical insurance. Dental services, unlike routine outpatient care, encounter more significant financial obstacles due to the inadequacy of insurance coverage (21). The cost of dental care is notably high and often beyond the financial means of a significant portion of the population in developing countries (22, 23). Given the current reimbursement policy, a considerable portion of basic dental care services can be covered through the personal medical savings account in UEBMI. Consequently, disparities in dental care utilization may exist, necessitating focused research and policy attention.

Gaining insights into the disparities among various healthcare modalities can assist health policymakers in formulating more targeted policy strategies.

In this study, there are three hypotheses that require validation:

1. The personal medical savings account is anticipated to exert distinct effects on the utilization of diverse healthcare service categories.
2. The personal medical savings account is expected to yield varying impacts on the proportion of total costs to different healthcare service categories.
3. The personal medical savings account is expected to exert diverse effects on the out-of-pocket payment ratio concerning different healthcare service categories.

The research findings are poised to effectively showcase the influence of the personal medical savings account on the utilization of distinct health care services and their respective costs. Additionally, the study will compare the differences in these effects across various health care services. These results can furnish valuable data support and evidence for refining strategies aimed at enhancing health insurance coverage.

## Methods

### Study design and study sample

We conducted a cross-sectional, secondary analysis utilizing data derived from Wave 3 in 2015 of the China Health and Retirement Longitudinal Study (CHARLS). CHARLS, initiated in 2011, is a nationwide longitudinal survey designed to represent mainland China’s residents aged 45 and above. The survey systematically collects high-quality, representative panel data covering a diverse array of topics, encompassing demographic characteristics, socioeconomic status, family dynamics, health, and healthcare. Employing a multistage probability sampling approach, individuals aged 45 and above across China were randomly selected in 2011, constituting a baseline survey with 10,000 households and 17,500 individuals. Subsequently, the same cohort was followed up every 2 years. Comprehensive details regarding sampling procedures and the study methodology of CHARLS have been extensively discussed in previous studies (24).

For this study, the inclusion criteria encompassed individuals aged ≥45 years with complete and relevant information.

### Outcome variables

To explore the impact of the personal medical savings account of basic medical insurance on the utilization of diverse healthcare services, the designated outcome variables for this phase encompassed “outpatient service utilization in the last month”, “inpatient service utilization in the last year”, “dental service utilization in the last year”, and “physical examination service utilization in the last 2 years.” Within the CHARLS questionnaire, specific items corresponding to each of these variables will be employed. These variables are binary in nature, represented by responses of “yes” or “no.”

To examine the influence of the personal medical savings account on the expenditure related to various healthcare services, the outcome variables in this stage were distinctly defined as the total expenditures for outpatient care, inpatient care, and dental care. Notably, the original questionnaire did not encompass inquiries regarding physical examination expenditure.

To examine the impact of the personal medical savings account on the out-of-pocket ratio for each type of healthcare service, the designated outcome variables for this stage were individually defined as the out-of-pocket fee divided by the total expenditure for outpatient care, inpatient care, and dental care.

A comprehensive description of each variable is provided in Supplementary Table S1.

### Independent variables

The independent variables were defined based on whether the respondents were enrolled in basic medical insurance. If a respondent chose any form of basic health insurance, they were categorized as participants in basic health insurance. Conversely, if they did not select any form of basic health insurance, it signified their non-participation in basic health insurance.

Simultaneously, the original questionnaire consolidated participation types. Respondents who individually selected URBMI, New Cooperative Medical Insurance, Urban and Rural Resident Medical Insurance, Medical Aid, or No Insurance were categorized as “participants without a personal medical account.” On the other hand, respondents who separately chose Urban Employee Medical Insurance, Government Medical Insurance, Private Medical Insurance (purchased by the work unit or the individual), Urban Non-Employed Person’s Health Insurance, and Other Medical Insurance were classified as “participants with a personal medical account.”

### Covariates

The selection of covariates was based on prior studies identifying factors that may influence healthcare utilization. These covariates encompassed gender, age, level of education, residential area, dental health indicated by edentulism, presence of comorbidities, annual medical insurance premium, self-assessed health status, and family financial status represented by bank deposits.

### Statistical methods

The participants were characterized to present demographic information, the enrollment rate in basic medical insurance, the rate of having a personal medical savings account, and other basic attributes. Subsequently, a Seemingly Unrelated Regression (SUR) approach was employed to analyze the disparities in the impact of personal medical savings account on various outcome variables.

The Seemingly Unrelated Regression (SUR), also known as the Seemingly Unrelated Regression Equations (SURE) model, was introduced by Arnold Zellner in 1962 (25). The SUR model is an extension of the linear regression or logit regression models and encompasses multiple regression equations, each with its own dependent variable and potentially distinct sets of exogenous explanatory variables. Each equation is an independent and valid regression, allowing separate estimation, hence the term “seemingly unrelated.” The SUR model can be regarded as a streamlined variant of the general linear model, involving specific coefficients in the parameter matrix *β* constrained to zero. Alternatively, it can be perceived as an expansion of the general linear model, allowing variations in the independent variables on the right-hand side across each equation. Moreover, the SUR model can be extended into the simultaneous equations model, where the right-hand side variables can also be considered endogenous. Widely utilized in econometrics and related disciplines, the SUR model serves to analyze and model intricate relationships between variables (26, 27). To examine the effects of covariates, odds ratios were reported from a logit SUR model, and average marginal effects were reported from a linear SUR model.

In the sensitivity analysis, the selection model introduced by Heckman was employed. The Heckman selection model, also referred to as the Heckit model, is a method utilized to estimate regression models afflicted by sample selection bias (28). Within the Heckman selection framework, the dependent variable is observable for only a portion of the dataset. A quintessential example in economics illustrating the sample selection predicament is the wage equation for women. In this scenario, a woman’s wage is observed only if she opts to enter the workforce; otherwise, it remains unobservable. Heckman’s seminal paper introducing the Heckman Selection model specifically addressed this challenge (28).

The coefficient for each variable and their respective 95% confidence intervals (95% CIs) were provided. Statistical significance was determined at a threshold of *p* < 0.05. All data were analyzed using STATA 14.0 (StataCorp, TX, USA).

## Results

### Sample descriptive analysis

A total of 15,628 individuals were included in the analysis after excluding 2,806 individuals with missing outcome variables (see Table 1). In the overall sample, the mean age was 61.8 ± 9.7, with 52.4% (*n* = 8,184) being female. Regarding health coverage, 95.5% (*n* = 14,927) were covered by basic medical insurance, and only 12.8% (*n* = 1,999) of individuals possessed a personal medical account.

**Table 1:** Sample descriptive information.

	*N*	Percent (%)
Total	15,628	100
Basic medical insurance		
Yes	14,927	95.5
No or other	701	4.5
Basic medical insurance types		
No medical insurance	701	4.5
Private medical insurance	35	0.2
Socialized medicine	258	1.7
URBMI	12,893	82.5
UEBMI	1741	11.1
Have personal medical account		
Yes	1999	12.8
No	13,629	87.2
Sex		
Male	7,444	47.6
Female	8,184	52.4
Age	61.8 ± 9.7	
Living area		
Urban area	2,877	18.4
Combination area	1,237	7.9
Rural area	11,514	73.7
Education level		
Primary school and below	7,098	45.4
Middle school	6,700	42.9
High school and above	1830	11.7
Deposit in bank	39213.2 ± 151622.7	
Self-reported health status		
Very good	869	5.56
Good	1,553	9.94
Fair	4,404	28.18
Poor	5,045	32.28
Very poor	2,309	14.77
Missing data	1,448	9.27
Edentulism		
Yes	2,476	15.8
No	13,152	84.2
Comorbidity situation		
≤1	7,896	50.5
>1	7,732	49.5
Yearly premium for basic medical insurance	183.0 ± 880.5	
Receive outpatient visit last month		
Yes	3,433	22.0
No	12,195	78.0
Total cost	1024.0 ± 3522.5	
Out-of-pocket paid ratio	0.84 ± 0.29	
Receive inpatient care last year		
Yes	1985	12.7
No	13,643	87.3
Total cost	15554.7 ± 22035.8	
Out-of-pocket paid ratio	0.52 ± 0.31	
Have dental visit last year		
Yes	2,687	17.2
No	12,941	82.8
Total cost	534.0 ± 1103.4	
Out-of-pocket paid ratio	0.97 ± 0.17	
Receive physical examination in recent 2 years		
Yes	6,363	40.7
No	9,265	59.3

URBMI: Urban Residents’ Basic Medical Insurance System; UEBMI: Urban Employee Basic Medical Insurance; Categorical variable was described as count number and percent; Continuous variable was described as mean ± stand deviation.

Among the total individuals, 22.0% of participants reported receiving outpatient care last month, 12.7% received inpatient care last year, 17.2% received dental care last year, and 40.7% underwent a physical exam in the last 2 years.

### Effect of basic medical insurance on healthcare utilization

Before SUR was employed, the correlation matrix along with *p* values for statistical significance was checked. *p* value of correlation matrix of residuals from separate regressions for outpatient, inpatient, dental, and physical examination services to illustrate the degree of correlation. All *p*-value < 0.05, the correlation is statistically significant, which supports SUR. Correlation matrix was show in Supplementary Table S5.

**Figure 1:**
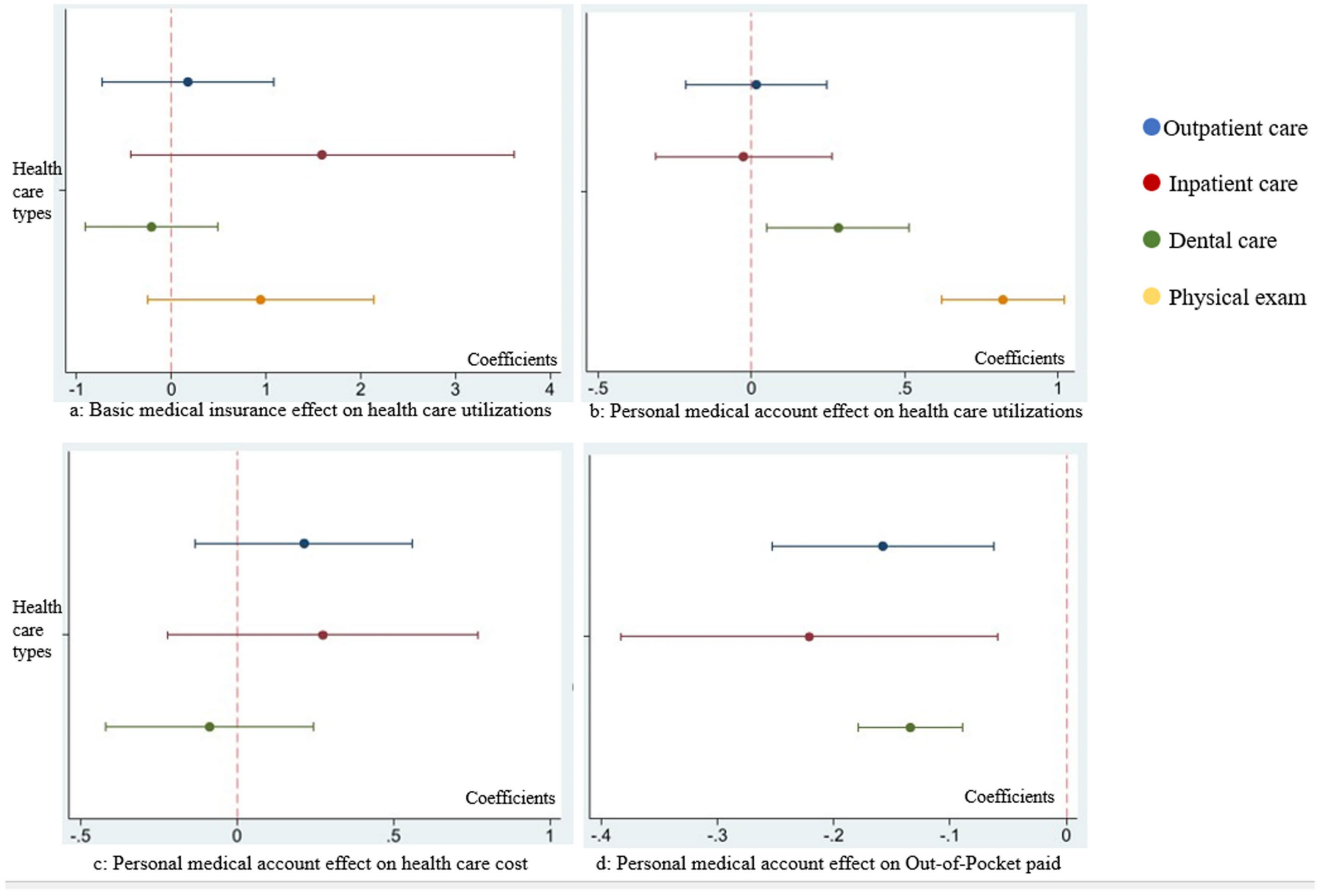
Comparison on coefficients of basic medical insurance and personal medical savings account in each seemingly unrelated regression. **(a)** Coefficients comparison of basic medical insurance effect on outpatient care, inpatient care, dental care, and physical exam utilizations. **(b)** Coefficients comparison of personal medical account effect on outpatient care, inpatient care, dental care, and physical exam utilizations. **(c)** Coefficients comparison of personal medical account effect on the expenditure of outpatient care, inpatient care, and dental care. **(d)** Coefficients comparison of personal medical account effect on out-of-pocket paid ratio of outpatient care, inpatient care, and dental care.

Seemingly unrelated logit regression was conducted to assess the impact of basic medical insurance on the utilization of outpatient care, inpatient care, dental visits, and physical exams. However, no significant differences were observed in various types of healthcare utilization. Figure 1a presents the coefficient of basic medical insurance, with detailed information provided in Supplementary Table S2.

### Effect of personal medical savings account on healthcare utilization

We analyzed the impact of having a personal medical account on the utilization of outpatient care, inpatient care, dental visits, and physical exams. The results from SUR analysis (Table 2) indicated that having a personal medical account was significantly associated with increased utilization of dental visits (OR: 1.327, 95% CI: 1.05–1.67) and a higher frequency of physical examinations (OR: 2.271, 95% CI: 1.86–2.77).

**Table 2:** Results of seemingly unrelated logit regression analyzing the impact of personal medical account on healthcare utilizations.

	Outpatient care utilization			Inpatient care utilization		
	Odds ratio	*p*-value	95%CI	Odds ratio	*p*-value	95%CI
Have personal medical savings account	1.016	0.893	[0.81–1.28]	0.975	0.863	[0.73–1.3]
Sex	1.258	0.005	[1.07–1.48]	1.021	0.843	[0.83–1.26]
Age	0.999	0.896	[0.99–1.01]	1.024	0.000	[1.01–1.04]
Education	0.986	0.836	[0.87–1.12]	0.930	0.391	[0.79–1.1]
Living area						
Combination area	0.904	0.538	[0.66–1.25]	0.915	0.670	[0.61–1.37]
Rural area	0.973	0.797	[0.79–1.2]	0.897	0.426	[0.69–1.17]
Edentulism	1.156	0.229	[0.91–1.46]	0.958	0.778	[0.71–1.29]
Comorbidity	1.263	0.000	[1.2–1.32]	1.349	0.000	[1.27–1.43]
Yearly premium	1.000	0.760	(1–1)	1.000	0.060	(1–1)
Self-reported health status	1.135	0.000	[1.06–1.21]	1.100	0.024	[1.01–1.2]
Deposit in bank	1.000	0.718	(1–1)	1.000	0.841	(1–1)
cons	0.089	0.000	[0.04–0.2]	0.014	0.000	[0–0.04]

	Dental visit			Physical examination		
	Odds ratio	*p*-value	95%CI	Odds ratio	*p*-value	95%CI
Have personal medical savings account	1.327	0.016	[1.05–1.67]	2.271	0.000	[1.86–2.77]
Sex	1.284	0.003	[1.09–1.52]	1.311	0.000	[1.14–1.51]
Age	1.002	0.653	[0.99–1.01]	1.037	0.000	[1.03–1.05]
Education	1.313	0.000	[1.15–1.5]	1.248	0.000	[1.12–1.4]
Living area						
Combination area	0.784	0.147	[0.56–1.09]	0.663	0.003	[0.51–0.87]
Rural area	0.868	0.198	[0.7–1.08]	0.752	0.002	[0.63–0.9]
Edentulism	1.584	0.000	[1.25–2.01]	1.187	0.108	[0.96–1.46]
Comorbidity	1.050	0.061	[1–1.11]	1.106	0.000	[1.06–1.16]
Yearly premium	1.000	0.077	(1–1)	1.000	0.149	(1–1)
Self-reported health status	1.015	0.662	[0.95–1.08]	1.001	0.960	[0.95–1.06]
Deposit in bank	1.000	0.349	(1–1)	1.000	0.001	(1–1)
cons	0.071	0.000	[0.03–0.16]	0.036	0.000	[0.02–0.07]

A visual comparison of the coefficients for personal medical account across the four types of healthcare utilization was illustrated in Figure 1b.

### Effect of personal medical savings account on healthcare expenditure

Seemingly unrelated negative binomial regression was performed to analyze the effect of personal medical account effect on outpatient care, inpatient care, and dental visit expenditure.

However, there were no significant difference among various kinds of health care expenditure. The effect comparison of personal medical savings account on health care expenditure in each equation was in Figure 1c. The detail information was in Supplementary Table S3.

### Effect of personal medical savings account on the out-of-pocket ratio of different healthcare expenditure

Seemingly unrelated linear regression was utilized to analyze the impact of a personal medical account on the out-of-pocket ratio for outpatient care, inpatient care, and dental visit expenditures. Having a personal medical savings account is associated with a statistically significant reduction in the out-of-pocket ratio of outpatient healthcare. Specifically, the average marginal effect (AME) is −0.158 (*p* = 0.001, 95%CI: −0.25 to −0.06), indicating that individuals with such accounts, on average, have a 15.8 percentage point lower out-of-pocket payment ratio compared to those without (see Table 3). Having a personal medical savings account is significantly associated with lower out-of-pocket ratios across multiple types of healthcare services. For inpatient care, the average marginal effect (AME) is −0.221 (*p* = 0.008; 95% CI: −0.38 to −0.06), indicating a 22.1 percentage point reduction in out-of-pocket payments. For dental services, the AME is −0.134 (*p* < 0.001; 95% CI: −0.18 to −0.09), while for outpatient services, the AME is −0.158 (*p* = 0.001). These findings suggest that individuals with personal medical savings accounts tend to bear significantly lower financial burdens across various health service types. The comparative effect of the personal medical account on the out-of-pocket ratio in each equation is presented in Figure 1d.

**Table 3:** Seemingly unrelated negative binomial regression of personal medical account effect on outpatient care, inpatient care, and dental visit health care expenditure.

	AME	*P*	95%CI
Out-of-pocket ratio of outpatient health service			
Have personal medical savings account	−0.158	0.001	[−0.25--0.06]
Sex	0.021	0.528	[−0.04–0.08]
Age	−0.006	0.005	[−0.01–0]
Education	−0.012	0.668	[−0.06–0.04]
Living area			
Combination area	−0.025	0.695	[−0.15–0.1]
Rural area	−0.023	0.591	[−0.11–0.06]
Edentulism	0.027	0.574	[−0.07–0.12]
Comorbidity	−0.001	0.914	[−0.02–0.02]
Yearly premium	0.000	0.684	[0–0]
Self-reported health	−0.011	0.381	[−0.04–0.01]
Deposit in bank	0.000	0.003	[0–0]
Out-of-pocket ratio of inpatient health service			
Have personal medical savings account	−0.221	0.008	[−0.38--0.06]
Sex	−0.010	0.853	[−0.12–0.1]
Age	0.000	0.940	[−0.01–0.01]
Education	0.010	0.832	[−0.08–0.1]
Living area			
Combination area	0.201	0.101	[−0.04–0.44]
Rural area	0.083	0.276	[−0.07–0.23]
Edentulism	−0.061	0.410	[−0.21–0.08]
Comorbidity	0.008	0.611	[−0.02–0.04]
Yearly premium	0.000	0.753	[0–0]
Self-reported health	−0.014	0.459	[−0.05–0.02]
Deposit in bank	0.000	0.873	[0–0]
Out-of-pocket ratio of dental visit			
Have personal medical savings account	−0.134	0.000	[−0.18--0.09]
Sex	−0.029	0.077	[−0.06–0]
Age	0.000	0.934	[0–0]
Education	−0.003	0.806	[−0.03–0.02]
Living area			
Combination area	−0.008	0.804	[−0.07–0.06]
Rural area	−0.029	0.186	[−0.07–0.01]
Edentulism	0.004	0.867	[−0.04–0.05]
Comorbidity	0.006	0.249	[0–0.02]
Yearly premium	0.000	0.238	[0–0]
Self-reported health	−0.003	0.675	[−0.02–0.01]
Deposit in bank	0.000	0.301	[0–0]

### Sensitivity analysis

The sensitivity analysis corroborated our primary findings. The Heckman selection model was employed to analyze the effect of the personal medical account on the selection and expenditure related to each type of healthcare utilization. The results indicated a significant association between the personal medical account and outpatient care as well as dental care utilization, while no association was observed with corresponding healthcare expenditure. Detailed information is provided in Supplementary Table S4.

## Discussion

This study revealed that variations in healthcare service utilization associated with different insurance types may be linked to the presence of personal medical savings accounts. Notably, individuals with personal medical savings accounts exhibited significantly higher utilization of oral health and physical examination services compared to those without such accounts. However, no significant differences were observed in the utilization of outpatient and inpatient services. Additionally, individuals with personal accounts incurred significantly lower out-of-pocket expenses across all types of medical services. This finding suggests a potential mechanism through which personal accounts influence healthcare service utilization.

A key aspect highlighted in this study is the disparity in healthcare service utilization between individuals with and without personal medical savings accounts. Unlike previous research that examined healthcare services separately (29), our study innovatively integrated dental care and physical examinations with outpatient and inpatient services, utilizing a seemingly unrelated regression approach. Our findings indicate that having a personal medical savings account is associated with increased utilization of dental and preventive care services. This pattern may be explained by the flexibility of personal medical savings accounts, which allow enrollees greater autonomy in healthcare decisions.

### The role of personal medical savings accounts in healthcare utilization

In China’s basic medical insurance system, personal medical savings accounts are primarily funded by employer contributions and can be used for outpatient expenses, including dental care. Unlike pooled funds, which are mainly allocated for inpatient or severe outpatient care and are subject to stricter reimbursement policies, personal accounts provide greater flexibility. The use of these funds is largely at the discretion of the individual, with minimal administrative restrictions, particularly regarding dental services, as long as expenses remain within the account balance.

According to the Fourth National Oral Health Survey (2015–2016), only 21.4% of adults (35–44 years old) and 20.7% of older adults (65–74 years old) utilized oral health services in the past 12 months (30). Despite the relatively low utilization, the financial burden of dental care remains high, with disproportionate out-of-pocket expenses regardless of whether payments come from personal funds or insurance coverage. Given that dental care often requires significant out-of-pocket spending and is not fully covered by pooled insurance funds, personal medical savings accounts may serve as an alternative funding source, facilitating greater access to these services. While not an explicit policy incentive, the autonomy in using personal accounts may function as an implicit driver of preventive and non-urgent care utilization.

### Insurance coverage and healthcare utilization disparities

Previous studies have identified disparities in healthcare service utilization across different insurance schemes, particularly in hospitalization rates (31). However, our study did not observe significant differences in outpatient or inpatient service utilization between individuals with and without personal medical savings accounts. This could be attributed to China’s high medical insurance coverage rate, which has exceeded 95%. Since most study participants were already insured under the basic medical insurance system, differences in service utilization were relatively minor.

While insured individuals may not always distinguish between different medical insurance types based on funding sources or coverage scope, they do experience differences in payment mechanisms. Dental care and physical examinations are generally not covered as basic healthcare services (32); rather, they fall under preventive care rather than treatment-based utilization. Personal medical savings accounts introduce an element of personal financial responsibility (33), which could explain why individuals with these accounts are more likely to use dental and preventive healthcare services. Given this dynamic, maintaining and expanding personal medical savings accounts within fiscal constraints could further encourage preventive healthcare utilization.

### Policy implications and considerations

The observed differences in out-of-pocket payment ratios may also reflect regional variations in policy implementation. In many provinces, personal accounts can be directly used for outpatient and dental expenses, whereas inpatient services are primarily covered by pooled funds and subject to stricter reimbursement policies. This regulatory distinction may explain why the financial protection effect of personal accounts is more pronounced for outpatient and dental services than for inpatient care.

Understanding the disparities associated with personal medical savings accounts is crucial for shaping public health insurance policies. In April 2021, the General Office of the State Council of China issued the “Guiding Opinions on Establishing and Enhancing the Outpatient Mutual Assistance Mechanism for Employees’ Basic Medical Insurance” (34), aiming to reform personal medical savings accounts by integrating outpatient mutual assistance mechanisms. This reform seeks to gradually include routine outpatient expenses for common illnesses under pooled fund reimbursements, thereby reducing the financial burden on insured individuals and promoting a more equitable and sustainable healthcare system.

However, while this policy change aims to optimize resource allocation, it may also exacerbate disparities between different insured groups. To address this challenge, policymakers should consider expanding personal medical savings accounts to cover a broader population, including those enrolled in the URBMI. Additionally, targeted measures—such as dedicated funding for dental and preventive healthcare services for individuals without personal accounts—could help bridge the gap between the two insured groups.

While personal medical savings accounts offer benefits in terms of enhanced access to outpatient and dental services, potential concerns must also be addressed. These include the risk of overutilization of non-essential services, unequal fund accumulation across income groups, and the potential depletion of account balances, particularly for individuals with chronic conditions. To balance equity and efficiency, policymakers should implement safeguards such as service eligibility criteria, coverage caps, and integration with broader risk-pooling mechanisms to ensure sustainable healthcare financing.

### Limitations and strengths

This study has certain limitations. Mainly, it is a cross-sectional analysis, and does not explore the changes in healthcare service utilization from a causal perspective caused by the changes in participation in basic medical insurance. Therefore, future research can further explore this aspect.

The other limitation of this study is that it focuses on individuals aged 45 and above, as determined by the CHARLS dataset. This age group is particularly relevant for examining dental care utilization, given the higher prevalence of dental problems and increased interaction with the healthcare system. However, the findings may not be fully generalizable to younger cohorts, who may face different barriers to care and hold different perceptions of personal medical accounts.

Future research including younger populations is warranted to explore age-related differences in dental service utilization and insurance behavior. From a policy perspective, age-stratified approaches may be needed—emphasizing preventive dental care in younger individuals and expanding coverage for restorative care among older adults.

Despite the aforementioned limitations, this study also analyzes different healthcare service utilization and out-of-pocket medical expense ratios from the perspective of having a personal medical savings account. Considering that basic medical insurance is already relatively stable among the middle-aged and older adult population in China, the nationally representative data reflects that there is still room for improvement in the development of basic medical insurance. This implies the continuous need to refine health insurance policies and vigorously promote the development of the basic economic level in order to reduce the disparities in healthcare service utilization among different insured groups.

## Conclusion

This study found that individuals who have a personal medical savings account in basic medical insurance tend to have higher utilization rates for dental visits and physical examinations among middle-aged and older adult individuals in China. Additionally, having a personal account can significantly reduce the proportion of out-of-pocket expenses for outpatient care, inpatient care, and dental treatment. Therefore, further reforms in health insurance policies should focus on maintaining the personal medical savings account and eliminating disparities among different insured groups.

## Data Availability

The original contributions presented in the study are included in the article/Supplementary material, further inquiries can be directed to the corresponding author.
