# Supplementary material for: The impact of personal medical savings accounts on healthcare utilization and out-of-pocket costs in public basic health insurance: a national cross-sectional study
Source: Front Public Health. 2025 Apr 30;13:1571746. doi: 10.3389/fpubh.2025.1571746 (PMC12075232; doi:10.3389/fpubh.2025.1571746)
Supplement: Supplementary file 1 [file Data_Sheet_1.PDF]

Table S1 Description of outcome variables and independent variables from original questionnaires

Variables type		Variable name	Data type	Original question
Outcome variable	(1)	Outpatient service utilization last month	Bivariate	ED001 In the last month have you visited a public hospital, private hospital, public health center, clinic, or health worker's or doctor's practice, or been visited by a health worker or doctor for outpatient care?<br>1. Yes<br>2. No
	(2)	Inpatient service utilization last year	Bivariate	EE003 Have you received inpatient care in the past year?<br>1. Yes<br>2. No
	(3)	Dental service utilization last year	Bivariate	EH001 In the past year, have you seen a dentist for dental care, including dentures?<br>1. Yes<br>2. No
	(4)	Physical examination service utilization last two years	Bivariate	EC001 When did you take the last physical examination in the last two years?<br>1. Didn't take physical examination last two years<br>2. Have physical examination last two years.
	(5)	Outpatient care expenditure	Continuous	How much did all the visits to [ED004 answer] cost during the last month?
	(6)	Inpatient care expenditure	Continuous	EE005 What was the medical cost for all the inpatient care you received during the past year? (Only include fees paid to the hospital, including ward fees but excluding wages paid to a hired nurse, transportation costs, and accommodation costs for yourself or family members.)
	(7)	Dental care expenditure	Continuous	EH003 What was the medical cost for all the dental care in the past year?
	(8)	Outpatient care self-	Continuous	ED007 1. Self-paid part ____ (ED007_1) Yuan

		paid ratio = (8) / (5)		[Brackets50/100/200/500/1000]<br>2. Didn't pay anything.
	(9)	Inpatient care self-paid ratio = (9) / (6)	Continuous	EE024 What was the total medical cost of hospitalization? (Only include the fees paid to the hospital, excluding the wage of hired nurse, the fare or rent, but including the ward fees.)
	(10)	Dental care self-paid ratio = (10) / (7)	Continuous	EH004<br>1. Self-paid part _______ (EH004_1) Yuan<br>2. Didn't pay anything
Independent variable	(11)	Have basic medical insurance or not	Bivariate	EA001 Are you the policy holder/primary beneficiary of any of the types of health insurance<br>listed below?<br>1. Urban employee medical insurance<br>2. Urban resident medical insurance<br>3. New cooperative medical insurance<br>4. Urban and rural resident medical insurance<br>5. Government medical insurance<br>6. Medical aid<br>7. Private medical insurance: purchased by work unit<br>8. Private medical insurance: purchased by individual<br>9. Urban non-employed persons's health insurance<br>10. Other medical insurance (specify) ____<br>11. No insurance
	(12)	Personal medical account	Bivariate	The respondent who chose 1, 5, 7, 8, 9, 10 in (11) were seen as having personal medical account.<br>The respondent who chose 2, 3, 4, 6, 11 in (11) were seen as having no personal medical account.

Table S2 Seemingly unrelated logit regression results of having basic medical insurance on different health care utilization

	Coefficient	SE	z	P>|z|	95%CI	
**Outpatient care last month**						
Have basic medical insurance	0.174	0.495	0.350	0.725	-0.796	1.145
Sex	0.229	0.083	2.760	0.006	0.066	0.391
Age	0.000	0.005	-0.090	0.930	-0.010	0.009
Education	-0.011	0.063	-0.180	0.859	-0.135	0.113
Living area						
Combination area	-0.106	0.162	-0.650	0.514	-0.423	0.212
Rural area	-0.035	0.096	-0.360	0.716	-0.222	0.153
Edentulism	0.145	0.120	1.210	0.225	-0.089	0.380
Comorbidity	0.234	0.025	9.500	0.000	0.185	0.282
Yearly premium for public basic medical insurance	0.000	0.000	0.280	0.782	0.000	0.000
Self-reported health status	0.126	0.036	3.540	0.000	0.056	0.196
Deposit in bank	0.000	0.000	-0.500	0.620	0.000	0.000
Constant	-2.603	0.659	-3.950	0.000	-3.895	-1.311
**Inpatient care last year**						
Have basic medical insurance	1.592	1.068	1.490	0.136	-0.501	3.685
Sex	0.019	0.104	0.180	0.854	-0.185	0.223
Age	0.024	0.006	3.980	0.000	0.012	0.036
Education	-0.077	0.080	-0.970	0.332	-0.234	0.079
Living area						
Combination area	-0.087	0.206	-0.420	0.673	-0.490	0.317
Rural area	-0.099	0.122	-0.810	0.419	-0.339	0.141
Edentulism	-0.033	0.147	-0.230	0.820	-0.321	0.254
Comorbidity	0.299	0.029	10.370	0.000	0.243	0.356
Yearly premium for public basic medical insurance	0.000	0.000	1.750	0.081	0.000	0.000

Self-reported health status	0.095	0.046	2.050	0.040	0.004	0.186
Deposit in bank	0.000	0.000	0.220	0.828	0.000	0.000
Constant	-5.847	1.168	-5.010	0.000	-8.135	-3.558
**Physical exam last two years**						
Have basic medical insurance	-0.211	0.382	-0.550	0.581	-0.960	0.538
Sex	0.263	0.071	3.720	0.000	0.124	0.401
Age	0.044	0.004	10.460	0.000	0.036	0.052
Education	0.355	0.055	6.470	0.000	0.247	0.462
Living area						
Combination area	-0.591	0.134	-4.400	0.000	-0.854	-0.328
Rural area	-0.599	0.083	-7.260	0.000	-0.761	-0.438
Edentulism	0.143	0.106	1.340	0.179	-0.065	0.351
Comorbidity	0.110	0.022	4.920	0.000	0.066	0.153
Yearly premium for public basic medical insurance	0.000	0.000	2.400	0.016	0.000	0.000
Self-reported health status	-0.001	0.027	-0.050	0.960	-0.054	0.051
Deposit in bank	0.000	0.000	3.750	0.000	0.000	0.000
Constant	-3.419	0.526	-6.500	0.000	-4.450	-2.389
**Dental visit last year**						
Have basic medical insurance	0.942	0.635	1.480	0.138	-0.303	2.187
Sex	0.244	0.085	2.890	0.004	0.078	0.410
Age	0.006	0.005	1.160	0.245	-0.004	0.015
Education	0.324	0.064	5.090	0.000	0.199	0.448
Living area						
Combination area	-0.318	0.164	-1.940	0.053	-0.640	0.004
Rural area	-0.266	0.094	-2.830	0.005	-0.450	-0.082
Edentulism	0.452	0.122	3.700	0.000	0.212	0.692
Comorbidity	0.051	0.026	1.990	0.047	0.001	0.102
Yearly premium for public basic medical insurance	0.000	0.000	-1.500	0.133	0.000	0.000
Self-reported	0.013	0.033	0.400	0.693	-0.052	0.078

health status						
Deposit in bank	0.000	0.000	0.970	0.330	0.000	0.000
Constant	-3.720	0.790	-4.710	0.000	-5.268	-2.173

Table S3 Seemingly unrelated negative binomial regression results of having personal medical account on different health care expenditure

	Coefficient	SE	z	P>|z|	95%CI	
**Outpatient care cost last time**						
Have personal medical account	0.215	0.293	0.730	0.464	-0.359	0.789
Sex	-0.157	0.222	-0.710	0.480	-0.592	0.278
Age	0.019	0.012	1.550	0.122	-0.005	0.043
Education	0.052	0.150	0.340	0.731	-0.242	0.345
Living area	-0.225	0.153	-1.470	0.141	-0.524	0.074
Edentulism	0.196	0.339	0.580	0.563	-0.468	0.859
Comorbidity	0.140	0.066	2.120	0.034	0.010	0.269
Yearly premium for public basic medical insurance	0.000	0.000	-3.850	0.000	0.000	0.000
Self-reported health status	0.137	0.073	1.870	0.061	-0.007	0.280
Deposit in bank	0.000	0.000	-2.260	0.024	0.000	0.000
Constant	5.509	1.047	5.260	0.000	3.457	7.560
**Inpatient care cost last time**						
Have personal medical account	0.275	0.298	0.920	0.356	-0.309	0.859
Sex	0.554	0.202	2.740	0.006	0.158	0.951
Age	-0.015	0.012	-1.270	0.203	-0.038	0.008
Education	0.126	0.127	0.990	0.323	-0.124	0.376
Living area	-0.075	0.148	-0.510	0.612	-0.364	0.214
Edentulism	0.481	0.305	1.580	0.114	-0.116	1.078
Comorbidity	-0.059	0.043	-1.360	0.175	-0.144	0.026
Yearly premium for public basic medical insurance	0.000	0.000	-1.660	0.096	0.000	0.000
Self-reported health status	0.092	0.062	1.490	0.136	-0.029	0.214
Deposit in bank	0.000	0.000	1.240	0.214	0.000	0.000
Constant	9.258	0.972	9.520	0.000	7.353	11.163
**Dental visit cost**						

	Coefficient	SE	z	P>|z|	95%CI	
Have personal medical account	-0.087	0.187	-0.470	0.641	-0.454	0.279
Sex	0.270	0.112	2.400	0.016	0.050	0.490
Age	0.015	0.008	1.840	0.065	-0.001	0.031
Education	0.250	0.100	2.500	0.012	0.054	0.446
Living area	-0.084	0.085	-0.990	0.324	-0.250	0.082
Edentulism	0.713	0.199	3.590	0.000	0.324	1.103
Comorbidity	-0.006	0.038	-0.160	0.873	-0.081	0.069
Yearly premium for public basic medical insurance	0.000	0.000	0.130	0.894	0.000	0.000
Self-reported health status	-0.038	0.050	-0.760	0.447	-0.136	0.060
Deposit in bank	0.000	0.000	1.570	0.116	0.000	0.000
Constant	4.667	0.626	7.460	0.000	3.441	5.893

Table S4 Heckman selection model result of having personal medical account on health care utilization and expenditure

	Effect on health care expenditure			Effect on health care selection		
Have personal medical account	β	*P* value	95% CI	β	*P* value	95% CI
Outpatient care	2880.71	0.784	-17739.59 - 23501.01	-0.20	<0.05	-0.36 - -0.02
Inpatient care	6692.90	0.735	-32104.42 - 45490.22	0.02	0.873	-0.23 - 0.27
Dental care	1402.80	0.835	-11759.87 - 14565.64	0.16	<0.05	0.04 - 0.30

Table S5 correlation matrix among outpatient\inpatient\physical exam\dental care

	Outpatient care	Inpatient care	Dental care	Physical exam
Outpatient care	1.0000			
Inpatient care	0.1563 *P*=0.0000	1.0000		
Dental care	0.0808 *P*=0.0000	0.0159 *P*= 0.0474	1.0000	
Physical exam	0.0819 *P*=0.0000	0.1119 *P*=0.0000	0.0592 *P*=0.0000	1.0000
